# Idiopathic Spontaneous Pneumomediastinum in an Active Patient With Crohn’s Disease

**DOI:** 10.7759/cureus.82256

**Published:** 2025-04-14

**Authors:** Jake A Cresta, Azfar S Syed, Jared S Magee, John G McCarthy

**Affiliations:** 1 Internal Medicine, Walter Reed National Military Medical Center, Bethesda, USA; 2 Gastroenterology, Tripler Army Medical Center, Honolulu, USA; 3 Gastroenterology, Walter Reed National Military Medical Center, Bethesda, USA

**Keywords:** acute complications of crohn's disease, crohn’s disease (cd), crohn's disease exacerbation, inflammatory bowel disease, pneumomediastinum

## Abstract

Spontaneous pneumomediastinum (SPM) is a rare complication of inflammatory bowel disease (IBD), often associated with colonic perforations or procedural interventions. This report details a case of a 30-year-old female with ileocolonic Crohn’s disease (CD) presenting with idiopathic SPM during an acute CD flare while on prolonged corticosteroid therapy. Imaging confirmed pneumomediastinum without evidence of esophageal or colonic perforation. Conservative management with supportive care and prophylactic antimicrobials led to clinical resolution. This case underscores the importance of recognizing SPM as a potential extraintestinal complication of CD, particularly in the context of prolonged steroid use.

## Introduction

Crohn's disease (CD) is a chronic inflammatory bowel disease (IBD) affecting any part of the gastrointestinal tract, with transmural inflammation and skip lesions. Its global prevalence is rising, typically starting between 15 and 30 years of age, and it is slightly more common in females. Unlike ulcerative colitis, which is limited to the colon, CD can affect the esophagus, stomach, and duodenum. It involves an overproduction of Th1 and Th17 cytokines, such as TNF-α, IL-12, and IL-23, driving inflammation. Anal complications, such as fistulas, occur in up to 50% of patients, often requiring surgery due to significant morbidity [1].

Spontaneous pneumomediastinum (SPM), defined as the unpredicted presence of free air in the mediastinum, is uncommon in the general population and exceedingly rare in the context of IBD. When SPM occurs with IBD, it is typically secondary to colonic perforations, toxic megacolon, or iatrogenic injury from preceding endoscopic procedures [2]. The pathophysiology is postulated to involve transmural inflammation or microperforations that allow air to track along fascial planes [3]. Corticosteroid use may further increase vulnerability by impairing tissue integrity and immune responses [4-6].

This case was previously presented as a meeting abstract at the 2024 American College of Gastroenterology (ACG) Annual Scientific Meeting on October 27, 2024.

## Case presentation

A 30-year-old active-duty female with a 10-year history of ileocolonic CD presented to our hospital with increasing lower abdominal pain, mixed bloody and mucoid diarrhea, and nocturnal bowel movements consistent with an acute CD flare. Her CD history had been complicated by the need for recurrent steroid tapers due to failure of several biologic agents, namely, adalimumab, infliximab, and ustekinumab. At presentation, she was nearly complete with a six-week prednisone taper following a recent hospitalization at an outside facility. She had also been experiencing globus, decreased appetite, and increased pyrosis after initiation of prednisone. She had received a loading dose of vedolizumab 300 mg once via intravenous infusion during her previous hospitalization six weeks prior, but she had not yet responded clinically. Due to conflicting work and travel obligations, she was not able to receive her next follow-up loading doses of vedolizumab prior to the current presentation.

Our patient works in public affairs with an unremarkable family history of IBD, colorectal cancer, or liver disease. She has no previous history of chronic lung disease, and she does not smoke cigarettes or vape. Her most recent hospitalization at an outside facility revealed a negative gastrointestinal infectious disease-related workup for her diarrhea. Her last colonoscopy more than two months prior showed anal stenosis and deeply ulcerated, serpiginous ulcers from the anal verge to the transverse colon.

In the emergency department, the patient appeared comfortable and was alert and oriented. Physical examination revealed stable vital signs, mild lower abdominal tenderness without peritoneal signs, normal respirations, and no subcutaneous crepitus. Laboratory test results are shown in Table 1. Laboratory findings were significant for acute leukocytosis and thrombocytosis. Additionally, her inflammatory markers were significantly elevated with a C-reactive protein (CRP) level of 9.4 mg/dL (normal range: <0.5 mg/dL) and erythrocyte sedimentation rate of 116 mm/hr (normal range: 2-37 mm/hr). Fecal calprotectin levels were 5930 mcg/g (normal range: <120 mcg/g).

**Table 1:** Laboratory data

Variable	Reference Range (Adults)	On Current Presentation
Hemoglobin (g/dL)	13.5-17.5	12.8
Hematocrit (%)	41.0-53.0	39.8
White-cell count (per µL)	4500-11,000	14,200
Platelet count (per µL)	150,000-400,000	605,000
Sodium (mmol/liter)	135-145	137
Potassium (mmol/liter)	3.4-5.0	4.7
Chloride (mmol/liter)	98-108	96
Carbon dioxide (mmol/liter)	23-32	27
Urea nitrogen (mg/dL)	8-25	14
Creatinine (mg/dL)	0.60-1.50	0.62
Calcium (mg/dL)	8.5-10.5	10.3
Lipase (U/liter)	0-160	18
C-reactive protein (mg/dL)	0-0.5	9.4
Erythrocyte sedimentation rate (mm/hr)	2-37	116
Fecal calprotectin (mcg/g)	0-120	5,930

**Figure 1:**
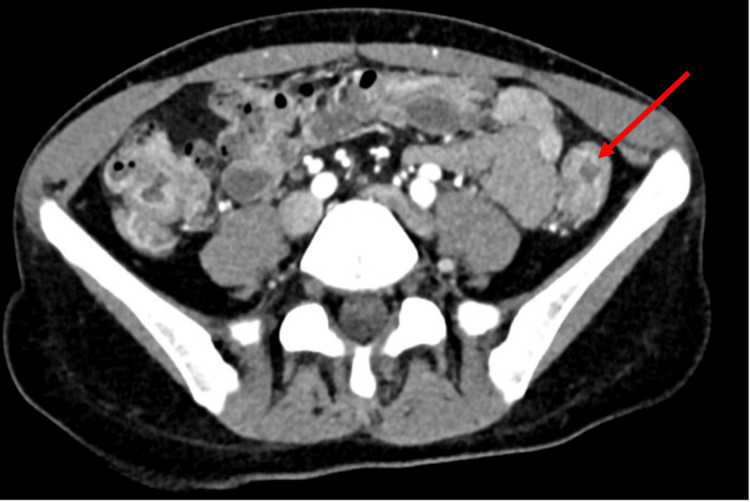
Computed tomography (CT) of the abdomen (axial), obtained with the administration of intravenous contrast material showing multifocal hyperemia and thickening of the colon, predominantly within the distal descending sigmoid (arrow). No signs of obstruction, perforation, or intra-abdominal fluid accumulation are present.

**Figure 2:**
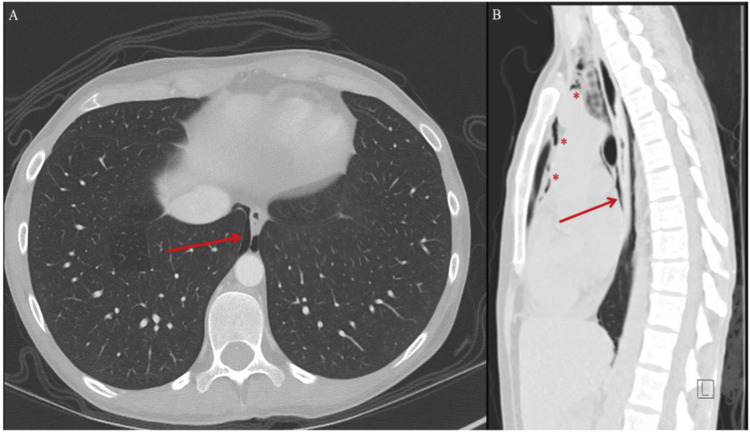
Computed tomography (CT) of the chest in axial (panel A) and sagittal (panel B) views obtained without the administration of oral or intravenous contrast material showing nonspecific pneumomediastinum, with contained air seen surrounding the lower esophagus (panels A and B, arrows). Free air is also seen extending along the anterior and superior mediastinum (panel B, asterisks).

**Figure 3:**
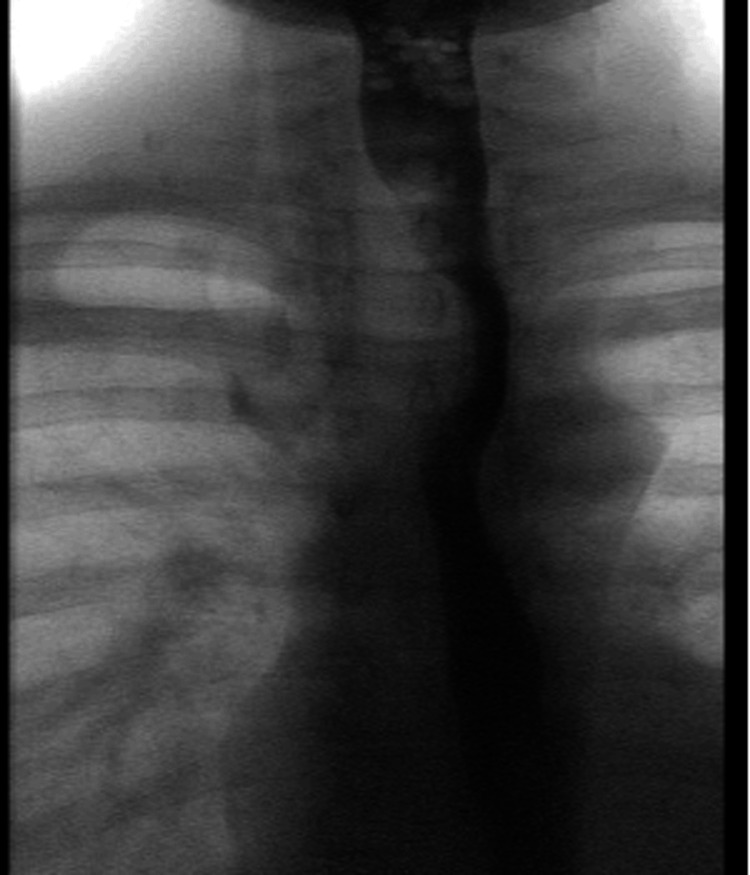
Gastrografin esophagram showing a normal esophagus without obstruction, delay, or evidence of perforation. There was no visible extravasation of contrast material.

Computed tomography (CT) imaging of the abdomen and pelvis revealed inflammatory changes of the rectosigmoid colon, consistent with IBD, without evidence of toxic megacolon or perforation (Figure 1). This imaging study also incidentally revealed pneumomediastinum with air surrounding the lower esophagus (Figure 2, panel A). A dedicated CT scan of the chest without contrast was immediately obtained, which confirmed the presence of air throughout the anterior and superior mediastinum (Figure 2, panel B). Otherwise, the lung parenchyma appeared normal without any evidence of interstitial lung disease, emphysema, or bronchiectasis. Gastrografin esophagram with oral contrast ruled out esophageal or colonic perforations (Figure 3).

The cardiothoracic surgery team was consulted and agreed with non-surgical management. Antimicrobial therapy consisted of a seven-day course of amoxicillin-clavulanic acid and fluconazole out of concern for mediastinitis, in conjunction with clinical observation. An induction dose of vedolizumab was administered prior to discharge from the hospital. The pneumomediastinum resolved clinically, and the patient’s IBD symptoms improved significantly.

Eight weeks after this episode, the patient’s fecal calprotectin decreased to 79 mcg/g, CRP normalized to 0.46 mg/dL, and the patient reported clinical remission. Plans were made for ongoing laboratory monitoring and repeat endoscopic evaluation to reassess for mucosal healing and ensure continued improvement.

## Discussion

Pneumomediastinum can arise from diverse etiologies such as blunt chest trauma, increased intrathoracic pressure leading to alveolar rupture, gas-forming infections, and iatrogenic perforations from procedures such as endoscopy [6]. However, SPM in the setting of IBD is rare and often linked to secondary causes such as colonic or esophageal perforation. In our case, no frank perforation was discovered on imaging studies, nor clinically suspected.

Symptoms of pneumomediastinum typically involve chest and neck discomfort, dyspnea, and dysphagia. Physical examination findings may include subcutaneous crepitus in the involved regions [3]. Another unique aspect of this case is the lack of an inciting endoscopic procedure, unlike most other reported cases [2,7-10]. Only several cases of SPM in the setting of IBD without a preceding endoscopic procedure have been described [3,11,12]. The absence of a toxic megacolon further differentiates this case from previously reported cases of SPM in the context of IBD [11,13].

The most accepted explanation for SPM supposes transient micro-perforations in the inflamed bowel wall during active CD that allow free air migration through retroperitoneal and diaphragmatic fascial planes into the mediastinum [2]. We postulate that our patient’s chronic corticosteroid use likely contributed to tissue fragility, predisposing the patient to SPM. Similar cases have implicated steroid-induced tissue vulnerability in the pathogenesis of pneumomediastinum, particularly in autoimmune conditions [4-6]. Data surrounding the time course or duration of corticosteroid use that predisposes development of SPM is poorly understood, given the variability of posited triggers and underlying conditions [14,15]. Ultimately, the causality between steroid use and SPM in this case remains unclear. Although data are limited, we also query whether a more gut-selective immunotherapy agent, such as vedolizumab, could be associated with these postulated microperforations.

Management strategies for SPM remain conservative in the absence of hemodynamic instability or overt perforation. This includes antibiotics, supportive care, and treating the underlying IBD flare [16]. Smoking cessation counseling, if applicable, should also be provided [17]. It has been suggested that an unrestricted diet can be advanced once symptoms have improved, provided that there are no complications such as esophageal perforation [18]. Both clinical and radiographic resolution of the pneumomediastinum may take up to several weeks.

## Conclusions

This case highlights the importance of maintaining a high index of suspicion for extraintestinal complications in patients with IBD. While corticosteroids clearly maintain a role in the acute management of IBD, clinicians should remain wary of the longstanding complications of prolonged corticosteroid use. More common complications of chronic corticosteroid use include opportunistic infections, metabolic and endocrinologic dysfunction, bone-mineral disease, and mood disturbances; however, we also suggest connective tissue weakening leading to perforation can lead to a rare, potentially devastating consequence such as SPM. Early recognition and appropriate management of SPM can prevent progression to life-threatening conditions. Moreover, it reinforces the importance of utilizing steroid-sparing agents in the long-term treatment of IBD.

## References

[1] Feuerstein JD, Cheifetz AS (2017). Crohn disease: epidemiology, diagnosis, and management. Mayo Clin Proc.

[2] Maunder RJ, Pierson DJ, Hudson LD (1984). Subcutaneous and mediastinal emphysema. Pathophysiology, diagnosis, and management. Arch Intern Med.

[3] Boshara P, Dalal BD (2023). A case of spontaneous pneumomediastinum associated with Crohn’s ileocolitis. Chest.

[4] Okamoto S, Tsuboi H, Noma H (2021). Predictive factors for pneumomediastinum during management of connective tissue disease-related interstitial lung disease: a retrospective study. Intern Med.

[5] Terasaki K, Okuyama Y, Ueda T, Matsuyama K, Urata Y, Yoshida N (2016). [A case report of severe ulcerative colitis with mediastinal and subcutaneous emphysema]. Nihon Shokakibyo Gakkai Zasshi.

[6] Subki AH, Almani IM, Albeity A, Aljabri BK, Alsolaimani R, Halabi H (2023). Spontaneous pneumomediastinum and subcutaneous emphysema in dermatomyositis: a case series and literature review. J Inflamm Res.

[7] Mihatov N, Fenves AZ (2015). Pneumomediastinum in inflammatory bowel disease. Proc (Bayl Univ Med Cent).

[8] Tam WC, Pollard I, Johnson RD (1996). Case report: pneumomediastinum and pneumothorax complicating colonoscopy. J Gastroenterol Hepatol.

[9] Hsu A, Higley C, Gupta B, Jennings JJ, Charabaty A (2019). Air everywhere: post-colonoscopy pneumomediastinum and pneumothorax in perianal Crohn’s disease. Gastrointest Endosc.

[10] Tai LC, Makarevich E, Alvarez A, Rubenstein I, Singh S (2024). Free air from where? Spontaneous pneumomediastinum and subcutaneous emphysema in a patient with ulcerative colitis in the absence of colonic or esophageal perforation. Inflamm Bowel Dis.

[11] Annaházi A, Polyák I, Nagy F, Wittmann T, Molnár T (2012). "Ulcerative crepitus" -- a case with subcutaneous emphysema and pneumomediastinum without colonic perforation or toxic megacolon in ulcerative colitis successfully treated conservatively. J Crohns Colitis.

[12] Cooke AA, Deshpande AV, Shun A, O'Loughlin EV (2010). Pneumomediastinum and subcutaneous emphysema in a child with ulcerative colitis. Pediatr Emerg Care.

[13] Mogan GR, Sachar DB, Bauer J, Salky B, Janowitz HD (1980). Toxic megacolon in ulcerative colitis complicated by pneumomediastinum: report of two cases. Gastroenterology.

[14] Barvaux VA, Van Mullem X, Pieters TH, Houssiau FA (2001). Persistent pneumomediastinum and dermatomyositis: a case report and review of the literature. Clin Rheumatol.

[15] Yamanishi Y, Maeda H, Konishi F, Hiyama K, Yamana S, Ishioka S, Yamakido M (1999). Dermatomyositis associated with rapidly progressive fatal interstitial pneumonitis and pneumomediastinum. Scand J Rheumatol.

[16] Alvares JF, Dhawan PS, Tibrewala S, Shankaran K, Kulkarni SG, Rananavare R, Kalro RH (1997). Retroperitoneal perforation in ulcerative colitis with mediastinal and subcutaneous emphysema. J Clin Gastroenterol.

[17] Gupta DC, Jenaw RK, Koolwal S, Khippal N (2014). A rare case of ulcerative colitis with diffuse parenchymal lung disease, spontaneous pneumomediastinum and subcutaneous emphysema. Indian J Chest Dis Allied Sci.

[18] Kim KS, Jeon HW, Moon Y, Kim YD, Ahn MI, Park JK, Jo KH (2015). Clinical experience of spontaneous pneumomediastinum: diagnosis and treatment. J Thorac Dis.

